# Progress in Disease of Digestive Organs

**Published:** 1901-07-20

**Authors:** 


### Progress in Disease of Digestive Organs.

(*Continued from page* 249.)

**Malignant Growths with Cirrhosis.**—The combination of carcinoma and cirrhosis has been pointed out by Finley, Adami, and others. Rolleston[46] reports another instance recently where the cells were formed into twisting tubules. He regards the growth as secondary to the cirrhosis, and as possibly due to a mis-development of the new bile ducts. William Hunter in discussing two other cases takes the view that the cancer arises by direct change of the liver cells themselves. Shattock points out that one class of hepatic cancer arises from the ducts and one from the cells. A. E. Thayer showed at New York[47] a specimen of carcinoma from a woman whose liver was also cirrhotic. The left lobe was nearly replaced by the neoplasm and the portal vein was invaded. He believed that the growth arose from the newly-formed bile ducts and had been carried along and distributed by the portal vein. *Sarcoma* with cirrhosis is still more rare. W. J. Ford[48] reports a primary mixed celled sarcoma, showing both round and spindle cells. This appears to be the first known instance of sarcomatous change in the connective tissue of a cirrhotic liver.

**Gall Stones.**—Jesse S. Meyer[49] holds that visceral ptosis may by interfering with the flow of bile lead to the formation of calculi when the mucous membrane is attacked by a catarrhal inflammation due to an ascending or descending infection. The cells thrown off together with the albuminous exudate and masses of bacteria cause the precipitation of the elements of calculi. M. A. Chauffard[50] writes warmly of the success obtained in preventing a return of biliary colic by the administration of one or two grammes each ot salicylate of soda and benzoate of soda daily with or without a similar quantity of Carlsbad salts. This treatment is employed for from 10 to 20 days every month, according to the severity of the attacks, and may be continued for more than a year. He advises also that one or two perles of Haarlem oil should be taken in the evening every 8 or 10 days. The results have been surprising and most satisfactory.

E. L. Opie[51] in discussing the relation of cholelithiasis to disease of the pancreas shows that impaction of a calculus near the orifice of the common duct may lead to violent pain and collapse, with death in 48 hours. Hæmorrhage into and around the pancreas and foci of fat necrosis may be found in such cases. In less acute instances death may occur after some weeks, and the pancreas may then be black and necrotic and lying in an abscess cavity. Finally, instead of these acute lesions, long continued obstruction of the duct may cause chronic inflammatory changes in the pancreas.

**Acute Yellow Atrophy.**—Two cases are reported by A. Hall.[52] The first was a young married woman, not pregnant, in whom the disease ran a fatal course in three weeks. The liver weighed 33 ounces and no leucin or tyrosin could be detected in the urine. The second was a male, aged 20, who died 39 days after the first symptom. There was no leucin or tyrosin in the urine and the liver weighed 33½ ounces. In neither case was there any history of fright or phosphorus poisoning. The cases appeared at first to be simple catarrhal jaundice, and the final stage was ushered in with drowsiness, coma, and in one person with severe vomiting. In the woman the liver dulness was not diminished till a week before death. In both patients ecchymoses were found after death, and in one melena and hæmatemesis occurred during life. No bacterial discovery, except the common B. proteus, was made.

G. H. Pearce[53] reports a case in an active, healthy farmer, aged 33. He felt unwell for a week, the temperature reaching 102°, the liver dulness being much lessened, and the abdomen tympanitic. The symptoms then showed intermission, but epistaxis, vomiting, and marked jaundice suddenly came on with other hæmorrhages and the patient died. C. T. Anderson[54] at Capetown observed an instance in a healthy nurse aged 20. She complained of headache on January 13th. There was a little vomiting on the 18th, followed by abdominal pain and tenderness. Bile was found in the urine and some leucin. On the 24th the pyrexia and other symptoms disappeared, but soon delirium, coma, and paralysis came on and she died on February 2nd. The liver weighed 50 ozs. and showed granular, fatty, and pigmentary degeneration. There was no evidence of phosphorus poisoning and not any trace of it in the vomit. A case of abscess of the liver following repeated attacks of dysentery was diagnosed by V. D. Harris and Macready,[55] chiefly through the presence in the urine of leucin, tyrosin, and cholesterin. Finally, œdema appeared over the lower ribs on the right side, and upon incision a large abscess was found and drained. The patient made a complete recovery. The abnormal constituents in the urine disappeared after the operation, having been present at least for ten weeks. There was no pyrexia throughout, and it was two years since the last attack of dysentery before the symptoms of abscess appeared.

**Dietetics.**—Fusel oil is said not to be the cause of the troubles following the use of bad whiskey. The active agent is the aldehydes, and especially furfural, which produces the throbbing headache. Similarly, nicotine has been credited with injurious effects, but the amount in tobacco is very small and chiefly destroyed by the heat. The bad effects, such as headache and giddiness, are probably due to pyridine and its allies, and the amount of these depends on the completeness of combustion. Thus there is less in the cigarette than in the pipe, and less in the pipe than in the cigar.[56] The great value of sugar as a food is brought out in an admirable paper by H. Willoughby Gardner.[57] The production of sugar in 1900 amounted to nearly 8,000,000 tons. Of this two-thirds was beet, the growth of which was largely due to the action of the first Napoleon. The consumption in this country of sugar amounts to about three ounces daily for every human being, which is far more than any other European nation consumes, and three times as much as even the French, Germans, and Dutch eat. The Americans only come near us in this habit, and our foes, the Boers, are also great sugar eaters. The increase in height, weight, and activity of English people in the last forty years have been ascribed to the enormous increase of sugar eating. It appears that sugar is very easily digested and absorbed, readily stored up in the body, readily utilised when required, and oxidised with little or no waste. Moreover, it shields the proteids from destruction, and can in some circumstances be stored up as fat for future use. Mosso showed that sugar quickly restored fatigued muscles. This is done with remarkable rapidity, so that extraordinary muscular labour can be performed if frequent supplies of sugar are given. The Germans found that in exhausting field manœuvres soldiers can do more with a large sugar ration, and in consequence both French and German soldiers now get an increased allowance. Gardner points out that it is a valuable food for the aged, for convalescents, for patients with wasting diseases, and for children. Of course, when there is a tendency to glycosuria, or in children to "mucous disease," care must be exercised. Probably in children it should be given with other food. In rheumatism the effects are doubtful, and in stout persons who suffer from gout it is clearly injurious, but in thin gouty patients there is little fear of harm. Of course in diabetes and obesity it must be eschewed.

46 Lancet, Jan. 19. 47 Med. Rec., April 27. 48 Pract., Feb. 49 Med. Rec. Feb. 50 La Semaine Med., Jan. 2. 51 Med. Rec., Jan. 12. 52 Quar. Rev., Feb. 53 Brit. Med. Jour., Nov. 10. 54 Brit. Med. Jour., loc. cit. 55 Lancet Dec. 15. 56 Lancet, May 11. 57 Brit. Med. Jour., April 27.

